# Non-Invasive Serum Amyloid A (SAA) Measurement and Plasma Platelets for Accurate Prediction of Surgical Intervention in Severe Necrotizing Enterocolitis (NEC)

**DOI:** 10.1371/journal.pone.0090834

**Published:** 2014-03-06

**Authors:** Kostan W. Reisinger, Boris W. Kramer, David C. Van der Zee, Hens A. A. Brouwers, Wim A. Buurman, Ernest van Heurn, Joep P. M. Derikx

**Affiliations:** 1 Department of Surgery, Maastricht University Medical Centre, and Nutrition and Toxicology Research Institute (NUTRIM), Maastricht, the Netherlands; 2 Department of Pediatrics, Maastricht University Medical Centre, and School for Oncology and Developmental Biology (GROW), School of Mental Health and Neurosciences, Maastricht, the Netherlands; 3 Department of Surgery, Wilhelmina Children’s Hospital, University Medical Centre, Utrecht, the Netherlands; 4 Department of Neonatology, Wilhelmina Children’s Hospital, University Medical Centre, Utrecht, the Netherlands; 5 Formerly Department of Surgery, currently Maastricht University Medical Centre, NUTRIM Institute, Maastricht, the Netherlands; University of Leuven, Belgium

## Abstract

**Objective:**

To evaluate the value of biomarkers to detect severe NEC.

**Summary Background Data:**

The time point of surgery in necrotizing enterocolitis (NEC) is critical. Therefore, there is a need for markers that detect severe NEC, because clinical signs of severe NEC often develop late. This study evaluated the value of biomarkers reflecting intestinal cell damage and inflammation to detect severe NEC.

**Methods:**

29 neonates with NEC were included. Two definitions of moderate versus severe NEC were analyzed: medical NEC (n = 12) versus surgical or fatal NEC (n = 17); and Bell stage II NEC (n = 13) versus stage III NEC (n = 16). Urinary intestinal fatty acid binding protein (I-FABP), serum amyloid A (SAA), C3a and C5a, and fecal calprotectin were measured. C-reactive protein (CRP), white blood cell count (WBC) and platelet count data were measured in blood.

**Results:**

In both definitions of moderate versus severe NEC, urinary SAA levels were significantly higher in severe NEC. A cut-off value of 34.4 ng/ml was found in surgical NEC versus medical NEC (sensitivity, 83%; specificity, 83%; LR+, 4.88 (95% CI, 1.37–17.0); LR−, 0.20 (95% CI, 0.07–0.60)) at diagnosis of NEC and at one day prior to surgery in neonates who were operated later on. Combination of urinary SAA and platelet count increased the accuracy, with a sensitivity, 94%; specificity, 83%; LR+, 5.53 (95% CI, 1.57–20.0); and LR−, 0.07 (95% CI, 0.01–0.48).

**Conclusion:**

Urinary SAA is an accurate marker in differentiating severe NEC from moderate NEC; particularly if combined with serum platelet count.

## Introduction

Necrotizing enterocolitis (NEC) is the most severe gastrointestinal disorder in neonates, affecting predominantly premature infants [1]. Initial treatment consists of discontinuation of enteral feeding, intravenous administration of broad spectrum antibiotics, and cardiopulmonary support. In case of perforation or severe clinical deterioration on medical treatment, resection of affected bowel is often the treatment of choice [2]. This group carries the highest mortality [3], and it is therefore essential to detect these severe cases at an early time point. In addition to clinical signs and symptoms, various plasma markers have been described to correlate with the severity of NEC, including serum amyloid A (SAA) [4], intestinal Fatty Acid Binding Protein (I-FABP) [5], [6], E-selectin [7], C5a [8] and IL-6 [9]. As venous puncture is a delicate and unfavorable procedure in neonates and a primary cause of anemia among preterm infants [10], [11], it is highly desirable to use non-invasive methods to discriminate neonates with severe NEC from those that can be treated conservatively. Non-invasive measurement for predicting NEC severity has only been described for I-FABP (14 kDa) [12], [13]. Another protein which is a candidate for non-invasive measurement for its small molecular size is the plasma marker SAA. SAA is an acute-phase protein (11.5 kDa), quickly synthesized by the liver and the kidneys upon induction by pro-inflammatory cytokines [14]–[16].

Calprotectin, a heterodimeric peptide, is released from the cytosol of neutrophils upon activation. Fecal calprotectin is a specific marker for neutrophil infiltrate in bowel mucosa. In intestinal inflammation, calprotectin is readily detectable in feces and plasma, making fecal calprotectin a suitable marker for NEC and possibly for NEC severity [12], [17], [18].

Complement activation has been linked to the pathogenesis of NEC [19]; plasma levels of C5a are associated with perforation and death in patients with NEC [8]. The complement activation products C3a and C5a are important in all three complement pathways. Their small molecular size (both 11 kDa) renders these proteins suitable as urinary markers of NEC severity.

This study evaluated the accuracy of non-invasive urinary and fecal measurement of markers reflecting intestinal cell damage and inflammation in addition to classical serum markers of inflammation in predicting disease severity of NEC. Furthermore, this study provided control values of non-invasive markers in premature neonates.

## Patients and Methods

### Ethics Statement

Written informed consent was obtained from both parents, and the study was approved by the medical ethical committee of Maastricht University Medical Centre, according to the revised version of the Declaration of Helsinki (October 2008, Seoul). The principles of good clinical practice (GCP) were followed during this study.

### Patients and Sample Collection

All patients with NEC in the neonatal intensive care units (NICU) at Maastricht University Medical Centre and Wilhelmina Children’s Hospital in Utrecht, between January 2008 and August 2010 were included into this study. In all patients, NEC was confirmed with the current golden standard of abdominal X-ray showing pneumatosis intestinalis (Bell stage II or higher, Table 1) [20]. In this study, medical NEC was defined as Bell stage II or higher without the need for surgery and not resulting in death. The following definitions of moderate versus severe NEC were used; first, a clinical definition: medical NEC versus surgical or fatal NEC; and second, a theoretical definition: Bell stage II NEC versus Bell stage III NEC. The theoretical definition is widely used, however the clinical definition may be more appropriate to evaluate the need for surgery, which is a clinical decision. The need for surgery was determined by a pediatric surgeon together with the attending neonatologist.

**Table 1:** Modified Bell’s criteria according to Walsh and Kliegman.

Stage	Clinical findings	Radiographic findings	Gastrointestinal findings
IA	Apnea and bradycardia,temperature instability	Normal or intestinal dilation, mild ileus	Gastric residuals, emesis, mild abdominal distention
IB	Same as above	Same as above	Bright red blood from rectum
IIA	Same as above	Intestinal dilation, ileus, pneumatosis	Grossly bloody stools, prominent abdominal distention, absent bowel sounds
IIB	Mild metabolic acidosis andmild thrombocytopenia	Widespread pneumatosis, ascites,portal-venous gas	Abdominal wall edema with palpable loops and tenderness
IIIA	Mixed acidosis, oliguria,hypotension, coagulopathy	Definite ascites, no free air	Generalized peritonitis, abdominal wall edema, erythema and induration
IIIB	Shock, deteriorationin laboratoryvalues and vital signs	Pneumoperitoneum	Same as stage IIIA

Adapted from Walsh and Kliegman [23].

Urine from all included neonates was collected at time of clinical diagnosis of NEC and daily until discharge from the NICU by placing a dental cotton roll (Henry Schein, Almere, the Netherlands) in the diaper of the neonate. Once the roll was filled with urine, it was placed in a sterile 5 mL syringe (Becton Dickinson, Oxford, United Kingdom), the urine was transferred into Micronic tubes (Micronic B.V., Lelystad, the Netherlands) and stored at −20°C until batch analysis. In all neonates, urine samples at time of diagnosis (D) were analyzed for the current study. It is unclear whether measurement at this single time point provides optimal accuracy or whether the accuracy changes with ongoing disease. Therefore, urine samples collected at two days before surgery (S-2) and one day before surgery (S-1) were also analyzed in neonates operated on two or more days following diagnosis. This method enabled to better assess the potential predictive value of markers for surgery in this cohort.

Stool samples were obtained at diagnosis and stored immediately at −20°C until batch analysis. All analyses were performed by one person after completion of patient inclusion, who was unaware of clinical outcome.

To determine control values of these biomarkers, 20 consecutive premature infants (matched with NEC infants for birth weight and gestational age at birth) admitted to the NICU at Maastricht University Medical Centre, without gastrointestinal symptoms were included. In these premature neonates serving as controls, a urine sample and a feces sample was collected at one week following birth. All control neonates were on full enteral feeding by that time and had not experienced feeding problems.

### Urinary I-FABP Measurement

Urinary I-FABP was measured using an in-house enzyme-linked immunosorbent assay (ELISA) that selectively detects human I-FABP (lower detection limit: 12.5 pg/ml).

### Urinary SAA Measurement

Urinary SAA was measured using a commercially available ELISA kit (lower detection limit 15.0 ng/ml), kindly provided by Hycult Biotechnology (Uden, the Netherlands).

### Urinary C3a and C5a Measurement

Urinary C3a and C5a were measured using commercially available ELISA kits (C3a, lower detection limit 250 pg/ml; C5a, lower detection limit 1,250 pg/ml), kindly provided by Hycult Biotechnology (Uden, the Netherlands).

### Fecal Calprotectin Measurement

After thawing of feces, 100 mg was weighed and added to 4.9 ml extraction buffer (0.1M Tris, 0.15 M NaCl, 1.0 M urea, 10 mM CaCl_2_·2H_2_O, 0.1 M citric acid, 0.5% BSA, pH 8.0) [21]. After 30 minutes shaking, 1 ml of suspension was centrifuged at 10,000 rpm for 20 minutes at 4°C and supernatant was aliquoted and stored at −20°C. Calprotectin concentration was measured in lysate using the commercially available calprotectin ELISA (lower detection limit 625 ng/ml), kindly provided by Hycult Biotechnology (Uden, the Netherlands). Fecal calprotectin concentration is given in µg calprotectin per gram feces.

### Conventional Blood Laboratory Tests

Classical markers of inflammation (C-reactive protein (CRP), white blood cell count (WBC) and platelet count) were determined as part of routine care by the departments of clinical chemistry and were collected from the medical records.

### Statistical Analyses

Normality was tested by Kolmogorov-Smirnov. Mann-Whitney *U* test was used for between group comparisons for continuous data. Pairwise comparisons were analyzed using Wilcoxon Signed Ranks test. Dichotomous variables were compared using Fisher exact test. All data are presented as median and range. Receiver operating characteristic (ROC) curves were used to calculate the accuracy of the studied markers predicting disease severity. The ideal cut-off value for diagnosing severe NEC was defined as the cut off value with maximum sum of sensitivity and specificity.

To determine the accuracy of combined markers detecting severe NEC, logistic regression analysis was performed and then plotted in receiver operator characteristic (ROC) curves. Overall diagnostic accuracy of combined markers was represented by the area under the curve (AUC). The best cut-off point of predicted probabilities (P) was defined as the cut-off point with maximum sum of sensitivity and specificity. To calculate the linear function describing all combinations of ideal cut-off values for combined markers in the detection of severe of NEC, the cut-off point (P) was used in the following equation: Ln(P/(1−P)) = B_0_+B_1_X_1_+B_2_X_2_; in which B_0_ represents the constant of the logistic regression analysis and B_1_ and B_2_ represent the logistic regression coefficients of SAA and platelet count, respectively. By calculating coordinates of intersections with the x- and y-axis, the linear function describing the cut-off line of SAA and platelet count could be determined.

Sample size was calculated as follows: in a previous study, mean I-FABP levels were 36 pg/nmol creatinine in infants with severe NEC compared with 5 pg/nmol creatinine in infants with mild NEC [12]. With α = 0.05 and 1−β = 0.80, this resulted in a minimal sample size of 12 patients per group.

Statistical analyses were performed with Prism 5.0 for Windows (GraphPad Software Inc. San Diego, CA) and SPSS 15.0 for Windows (SPSS Inc. Chicago, IL). STARD statement for reporting studies of diagnostic accuracy was used in this study [22].

## Results

### Patients

Twenty-nine consecutive neonates (11 males) were included. Median gestational age was 30+5 [range: 26+2–38+2] weeks, median birth weight was 1400 [860–1960] g. Two definitions of moderate versus severe NEC were used; medically treated NEC (medical NEC group n = 12) versus NEC requiring operation or causing death (operative/fatal NEC group, n = 17), and NEC stage II (NEC II group, n = 13) versus NEC stage III (NEC III group, n = 16), based on the modified Bell’s staging criteria according to Walsh and Kliegman [23] (Table 1).

There were no significant differences in birth weight and sex between the medical NEC and operative/fatal NEC groups (Table 2A), or between the NEC II and NEC III groups (Table 2B). Gestational age however was significantly lower in the operative/fatal NEC group compared with the medical NEC group and in the NEC III group compared with the NEC II group.

**Table 2:** Baseline characteristics.

A. Medical NEC vs. operative/fatal NEC				
	Medical NEC (n = 12)	Operative/fatal NEC (n = 17)	Total	*P*
Gestational age (days)*	32+4 (26+2–38+2)	29+3 (27+2–32+6)	30+5 (26+2–38+2)	<0.05
Birth weight (grams)*	1465 (860–1960)	1288 (1000–1738)	1400 (860–1960)	0.15
Sex	7 M (58%)	4 M (24%)	11 M (38%)	0.12
**B. NEC II vs. NEC III**				
	**NEC II (n = 13)**	**NEC III (n = 16)**	**Total**	***P***
Gestational age (days)*	32+4 (26+2–38+2)	29+1 (27+2–32+6)	30+5 (26+2–38+2)	<0.05
Birth weight (grams)*	1465 (860–1960)	1220 (1000–1738)	1400 (860–1960)	0.17
Sex	7 M (54%)	4 M (25%)	11 M (38%)	0.14

*Data are presented as median (range).

Fifteen patients were operated (all underwent laparotomy with intestinal resection), 9 on the day of diagnosis. The other 6 patients underwent surgery on median day 3 [2]–[29] (late surgery); 2 on day 2, 2 on day 3, one on day 7 and one on day 29. Indication for surgery was NEC in all cases (5 perforation, 10 clinical deterioration), and mortality (5 infants) was always related to NEC or its sequelae, which was confirmed by a pathologist specialized in pediatric gastroenterology. Two patients died without being operated. Of the 6 patients undergoing late surgery, the indication for surgery was clinical deterioration.

### Urinary I-FABP

Urinary I-FABP levels were not statistically different between the operative/fatal NEC group (5,000 [1,100–1,712] pg/ml) and the medical NEC group (5,500 [300–142,000] pg/ml), *p* = 0.51, Table 3A. Similar results were found in the NEC III group (11,100 [1,100–1,712,000] pg/ml) compared with the NEC II group (4,800 [300–142,000] pg/ml), *p* = 0.41, Table 3B.

**Table 3:** Values of IFABP, calprotectin, CRP and WBC at diagnosis.

A. Medical NEC vs. operative/fatal NEC			
	Medical NEC (n = 12)	Operative/fatal NEC (n = 17)	*P*
I-FABP (pg/ml)	5,500 (300–142,000)	5,000 (1,100–1,712,000)	0.51
Fecal calprotectin (µg/g feces)	375 (146–848)	479 (108–684)	0.87
CRP (mg/l)	52.0 (7.0–267)	108 (1.0–245)	0.47
WBC (×10^9^ cells/l)	11.8 (3.4–18.1)	6.4 (3.0–22.1)	0.09
**B. NEC II vs. NEC III**			
	**NEC II (n = 13)**	**NEC III (n = 16)**	***P***
I-FABP (pg/ml)	4,800 (300–142,000)	11,100 (1,100–1,712,000)	0.41
Fecal calprotectin (µg/g feces)	378 (317–848)	495 (108–684)	0.75
CRP (mg/l)	53.5 (7.0–267)	128.5 (1.0–245)	0.51
WBC (×10^9^ cells/l)	10.5 (3.4–18.1)	7.1 (3.0–22.1)	0.19

Data are presented as median (range).

To investigate whether urinary I-FABP levels increased between time of diagnosis (D) and time of surgery, samples taken at two days (S-2) and one day (S-1) prior to surgery were analyzed. However, there were no significant differences.

### Urinary SAA

**Figure 1:**
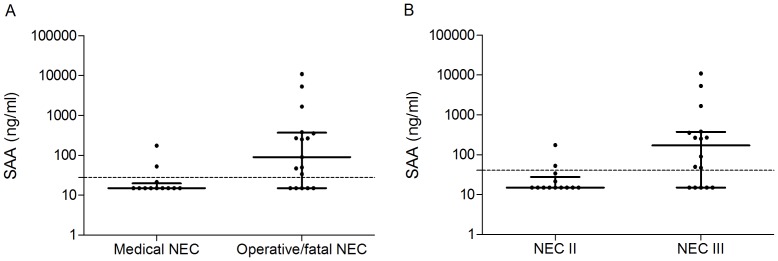
A. Urinary SAA levels at diagnosis (D) in the medical NEC group compared with the operative/fatal NEC group. The dotted line represents the ideal cut-off value of 27.8 ng/ml. B. Urinary SAA levels at diagnosis (D) in the NEC stage II group compared with the NEC stage III group. The dotted line represents the ideal cut-off value of 40.7 ng/ml.

Urinary SAA levels were significantly higher in the operative/fatal NEC group (90.8 [15.0–10,925] ng/ml) compared with the medical NEC group (15.0 [15.0–176] ng/ml), *p* = 0.007 (Figure 1A). Test characteristics are summarized in Table 4.

**Table 4:** Test characteristics of urinary SAA and platelet count.

Cut-off value	Sensitivity (%)	Specificity (%)	LR+ (95% CI)	LR− (95% CI)	AUC (95% CI)
SAA >27.8 ng/ml (D)	71	83	4.18 (1.15–15.0)	0.35 (0.16–0.77)	0.78 (0.61–0.95)
SAA >34.4 ng/ml (D/S-1)	83	83	4.88 (1.37–17.0)	0.20 (0.07–0.60)	0.87 (0.73–1.00)
Platelets <273×10^9^ cells/l (D)	76	83	4.47 (1.24–16.0)	0.29 (0.12–0.70)	0.75 (0.57–0.94)
Platelets <267×10^9^ cells/l(D/S-1)	76	83	4.47 (1.24–16.0)	0.29 (0.12–0.70)	0.78 (0.61–0.96)
SAA+platelets (D/S-1)*	94	83	5.53 (1.57–20.0)	0.07 (0.01–0.48)	0.93 (0.81–1.04)

*Cut-off line described by the linear function: [platelet count (10^9^ cells/l)] –25 • [SAA (ng/ml)] <159.3.

D = at diagnosis.

D/S-1 = at diagnosis (D) of neonates with moderate NEC, who were operated on the same day or who died; pooled with levels at one day prior to surgery (S-1) in neonates who were operated after the day of diagnosis.

LR+ = positive likelihood ratio.

LR− = negative likelihood ratio.

AUC = area under the curve.

CI = Confidence interval.

Equivalent results were found in the NEC III group (172 [15.0–10,925] ng/ml) compared with the NEC II group (15.0 [15.0–167] ng/ml), *p* = 0.01 (Figure 1B).

### Urinary SAA Preoperatively

**Figure 2:**
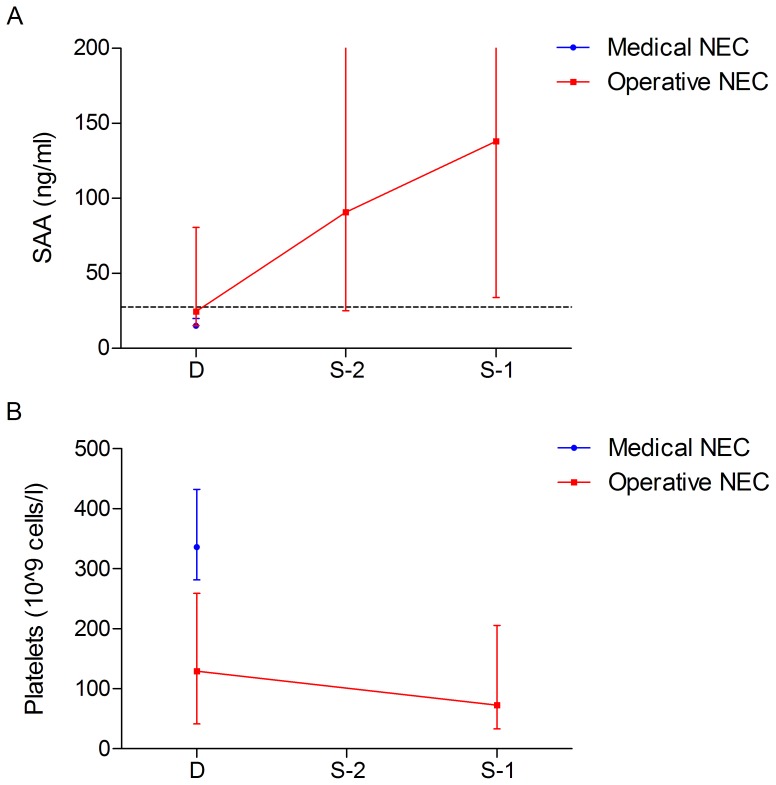
**A.** Development of urinary SAA levels in patients undergoing surgery 2 or more days after diagnosis. D: day of diagnosis, S-2∶2 days prior to surgery; S-1∶1 day prior to surgery. A cut-off value of 27.8 ng/ml is depicted by the dotted line. **B.** Development of platelet levels in patients undergoing surgery 2 or more days after diagnosis. D: day of diagnosis, S-2∶2 days prior to surgery; S-1∶1 day prior to surgery.

To investigate whether urinary SAA levels increased between time of diagnosis (D) and time of surgery, samples taken at two days (S-2) and one day (S-1) prior to surgery were analyzed. In the six neonates undergoing surgery after the day of diagnosis, urinary SAA levels did not change in the period between diagnosis (15.0 [15.0–28.0] ng/ml) and S-2 (35.2 [15.0–1,718] ng/ml, *p* = 0.66). SAA levels tended to be higher at S-1 compared with SAA levels at diagnosis (respectively, 15.0 [15.0–28.0] ng/ml and 138 [15.0–3,647] ng/ml, *p* = 0.06) although not reaching statistical significance, possibly caused by the small subgroup size (Figure 2A). At S-1, 4 out of 6 neonates had elevated urinary SAA levels, above the threshold of 27.8 ng/ml.

To assess whether the predictive value for surgery of urinary SAA changed with ongoing disease, urinary SAA levels at one day prior to surgery (S-1) were pooled with urinary SAA levels at diagnosis (D) of the neonates operated on the same day and neonates that eventually died of NEC. The area under the ROC curve in differentiating operative/fatal NEC from medical NEC increased to 0.87, test characteristics are summarized in Table 4). Similar results were found in differentiating the NEC III group from the NEC II group (data not shown).

### Urinary C3a and C5a

Urinary C3a and/or C5a levels were only detectable in 6 patients. Remarkably, these neonates were in the operative/fatal NEC and NEC III groups, however no reliable statistical differences could be calculated as complement activation products were not detectable in the majority of urine samples.

To investigate whether urinary C3a and C5a levels increased between time of diagnosis (D) and time of surgery, samples taken at two days (S-2) and one day (S-1) prior to surgery were analyzed. C3a levels increased in one patient prior to surgery and decreased in another patient prior to surgery. C5a levels were undetectable in samples at S-2 and S-1.

### Fecal Calprotectin

Fecal calprotectin levels analyzed in 16/29 patients who produced stools at diagnosis were not statistically different between the operative/fatal NEC group (479 [108–684] µg/g feces) and the medical NEC group (375 [146–848] µg/g feces, *p* = 0.87). Similar results were found for NEC stage III versus NEC stage II, Table 3.

### Conventional Blood Laboratory Tests

Values of C-reactive protein (CRP), white blood cell count (WBC), and platelets at the day of NEC diagnosis and one day prior to surgery were obtained from the patients’ medical records. No statistical differences between moderate and severe NEC were found for CRP and WBC, Table 3. At diagnosis, platelets were significantly decreased in operative/fatal NEC (204 [8–654]×10^9^ cells per liter) compared with medical NEC (336 [71–583]×10^9^ cells per liter, *p* = 0.02), and in NEC III (222 [8–654]×10^9^ cells per liter) compared with NEC II (328 [71–583], *p* = 0.05). Low platelet count yielded acceptable diagnostic accuracy in detecting severe NEC (test characterstics are summarized in Table 4). Similar results were found for NEC stage III versus NEC stage II.

Platelet levels in neonates operated several days after diagnosis dropped significantly from the day of diagnosis (130 [8–654]×10^9^ cells per liter) to the day prior to operation (73 [8–411], *p* = 0.03), Figure 2B. Platelet levels at one day prior to surgery (S-1) of neonates operated several days after diagnosis were pooled with platelet levels at diagnosis (D) of the neonates operated on the same day and neonates that eventually died of NEC. The area under the ROC curve in differentiating operative/fatal NEC from medical NEC did not change: 0.78 (test characteristics in Table 4). Results for NEC stage III versus NEC stage II were similar.

### Combination of Urinary SAA and Platelet Count in the Detection of Severe NEC

**Figure 3:**
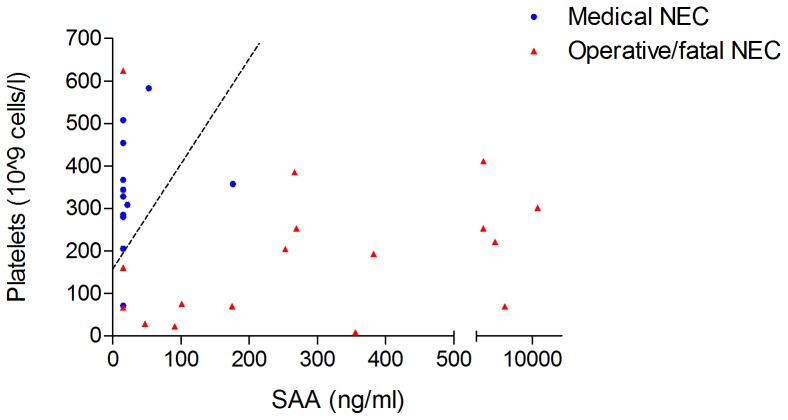
Combination of urinary SAA levels and platelet count in neonates with operative/fatal NEC (squares) and those with medically treated NEC (triangles) at diagnosis and one day prior to surgery in six neonates operated more than two days following diagnosis. The ideal cut-off line for differentiating between medical and operative/fatal NEC is depicted by the dotted line.

A logistic regression analysis approach was used to analyze the combination of urinary SAA with platelet count. For this analysis, the pooled dataset (D+S-1) was used. A cut-off line was calculated with area under the ROC curve, 0.93 (other test characteristics are described in Table 4), thereby increasing diagnostic accuracy compared to single marker measurement of urinary SAA or platelet count. The cut-off line was described by the linear function:

[platelet count (10^9^ cells/l)]–25×[SAA (ng/ml)] = 159.3; which means that a positive test is obtained when any combination of SAA and platelet levels in this formula results in a value <159.3 (Figure 3).

### Control Values of Markers

Control values of the urinary markers I-FABP, SAA, C3a and C5a and the fecal marker calprotectin were measured in 20 premature neonates admitted for prematurity and/or small for gestational age. The control values are listed in Table 5. Urinary SAA levels were not above the lower detection limit in all premature control neonates. C3a and C5a levels were only above the lower detection limit in respectively two controls and one control.

**Table 5:** Control values of urinary and fecal marker.

	Median (range)	Mean (SD)
I-FABP (pg/ml)	365 (174–924)	441 (234)
SAA (ng/ml)	No samples above lower detection limit of 15 ng/ml	
C3a (pg/ml)	250 (250–1570)*	
C5a (pg/ml)	1250 (1250–1473)**	
Calprotectin (µg/g feces)	68 (31–335)	110 (104)

*No samples except two above lower detection limit.

**No samples except one above lower detection limit.

## Discussion

This study shows that urinary levels of serum amyloid A (SAA) are significantly elevated in neonates with severe NEC defined as operative, fatal or stage III NEC compared with those with mild NEC, defined as medical or stage II NEC. Non-invasive measurement of SAA discriminates severe NEC from milder cases, especially in progressing disease, in which cases surgery was ultimately chosen for. Recently, Ng *et al.* reported that plasma SAA is a promising marker for the detection of NEC [16]. Furthermore, SAA in plasma has been reported to positively correlate with NEC stage [4], [24], [25]. In these studies, only the difference between Bell stages was investigated. Cetinkaya *et al.* (2010) showed that 80% of neonates with Bell stage I and 100% of neonates with Bell stage II/III had elevated plasma SAA levels [4]. This group also showed that plasma SAA levels were significantly higher in Bell stage III compared with Bell stage I at time of NEC diagnosis and on the days following diagnosis [24]. It may be questionable whether Bell stage I represents actual NEC, as no intestinal pneumatosis is present in this stage, however the results indicate an association between SAA levels and NEC stage. Eras *et al.* found elevated SAA levels in plasma in higher Bell stages at time of NEC diagnosis [25]. Urinary SAA has not been used previously to assess the severity of NEC. Non-invasive tests are favorable to minimize the risk of anemia [26]. Urinary measurement of SAA produces acceptable accuracy to detect severe NEC, and is able to differentiate between Bell stage II and Bell stage III. Serum platelet count markedly increases diagnostic accuracy, this procedure however necessitates blood sampling. In concordance with these results, low platelet count (less than 100×10^9^ cells/l) has been described before to correlate with extension of NEC [27] and laparotomy [28]. Platelet count alone resulted however in a post-test probability of 87% with a positive test and 29% with a negative test in the current study (pre-test probability of severe NEC, 59%). When combined, platelet count and urinary SAA yielded a post-test probability of 89% with a positive test and 9% with a negative test, indicating that the negative predictive value of platelet count is improved considerably when SAA is added.

Selection of severe NEC cases is important to determine whether surgical intervention should be considered. While most studies focus on a difference between Bell stages, the current study also investigated a difference between medically treated NEC and surgically treated NEC/fatal NEC, which may be more accurate to assess the required treatment. Expectedly, analyses of both definitions showed equivalent results. It must be noted that severe NEC versus mild NEC is only a surrogate definition suggesting which infants with NEC should undergo surgical intervention. Clear criteria for surgical intervention, i.e. a gold standard, are lacking. In addition, the optimal type of surgery is uncertain, since both peritoneal drainage and laparotomy with resection of affected gut result in equal mortality rates and other clinical outcomes [29].

The current study shows that early urinary SAA measurement correlates well with the clinical decision to perform surgery, and may therefore be able to assist the decision to perform surgery at an earlier time point. Future studies should focus on the effectiveness of early intervention as this matter has not yet been clarified. The median gestational age in the current study of 30+5 was higher compared to a recent study by Ng *et al.*: 28+3 [30], as was median birth weight: 1400 g compared to 1140 g [30]. The external validity of the current findings should therefore be verified in other hospitals, preferably in a multi-centered study design.

Analyses of other inflammatory markers in the current study showed no statistical differences between mild and severe NEC. However, the presence of elevated complement activation products (C3a and C5a) was limited to the severe NEC group, supporting the idea of an excessive inflammatory response in this group. Urinary measurement of C3a and C5a is not clinically relevant as these markers lacked diagnostic accuracy. Local inflammation as reflected by fecal calprotectin levels was not different in the severe NEC group compared with the mild NEC group. Moreover, no differences in intestinal epithelial cell damage reflected by I-FABP were observed between the groups. It can be hypothesized that severe NEC is characterized by increased systemic inflammation rather than increased mucosal breakdown and intestinal cell damage. It should be noted that fecal testing in NEC is not preferable as timing of bowel movements in preterm infants is unreliable and many patients present with (septic) ileus. In an earlier study by our group, urinary I-FABP levels accurately discriminated NEC from other diseases in neonates clinically suspected of NEC [12]. Therefore urinary I-FABP may be used to accurately diagnose NEC, while urinary SAA may help to guide the best treatment at an early stage of the disease, as an adjunct to clinical signs and symptoms. Serum platelet count may increase the accuracy of the SAA measurement.

A limitation of the study is the rather small group size. However to the best of our knowledge, this is one of the largest NEC cohorts, since definite NEC (Bell stage II and III) has a low incidence. Furthermore, this study represents two qualified pediatric surgery centers. Some remarks on the interpretation of the results of the current study must be made. First, urinary SAA measurement does not provide a 100% accuracy in selecting severe NEC cases. In the current cohort, with the given likelihood ratios and severe NEC prevalence of 59%, the post-test probability is 87% (89% when platelet count is added) after a positive test and 22% (9% when platelet count is added) after a negative test, meaning that urinary SAA measurement cannot completely rule out nor identify severe NEC. Second, all markers except the conventional inflammatory markers were measured after patient enrollment. Therefore, low platelet count could have been used to guide the clinical decision of performing surgery, and these findings should thus be taken with caution. Blinding of the clinician to conventional blood tests is however not ethical. Third, next to surgical NEC cases, infants who died from NEC without undergoing surgery were included in the severe NEC group as well. Since these were only two patients, separate statistical analysis was not possible. It remains to be determined whether these patients would have benefited from surgical intervention, which is only feasible in a prospective study. Fourth, clinical decisions cannot be based exclusively on a single or several biomarkers. However the use of biomarkers can be a useful tool when combined with clinical assessment, especially when clinical improvement or deterioration is doubted.
